# Bottom-up estimates of deep decarbonization of U.S. manufacturing in 2050

**DOI:** 10.1016/j.jclepro.2021.129758

**Published:** 2022-01-01

**Authors:** Ernst Worrell, Gale Boyd

**Affiliations:** a Copernicus Institute of Sustainable Development, Utrecht University, Princetonlaan 8A, 3584, CB, Utrecht, the Netherlands; b Social Science Research Institute, Department of Economics, Duke University, 140 Science Drive, Durham, NC, USA

**Keywords:** Climate change mitigation, Decarbonization, Energy efficiency, Electrification, Renewables, Material efficiency

## Abstract

The world needs to rapidly reduce emissions of carbon dioxide (CO_2_) emission to stave off the risks of disastrous climate change. In particular, decarbonizing U.S. manufacturing industries is particularly challenging due to the specific process requirements. This study estimates the potential for future CO_2_ emission reductions in this important sector. The analysis is a detailed accounting exercise that relies on estimates of emission-reduction potential from other studies and applies those potentials to the manufacturing sector using a bottom-up approach. The actions are grouped into four “pillars” that support deep decarbonization of manufacturing (DDM): Energy Efficiency, Material Efficiency, Industry-Specific, and Power Grid. Based on this bottom-up approach, the analysis shows that an 86% reduction in carbon dioxide emissions from the Reference Case is feasible. No single pillar dominates DDM, although opportunities vary widely by sub-sector. The analysis shows that a strategy incorporating a broad set of elements from each pillar can be effective instead of relying on any single pillar. Some pillars, such as Energy Efficiency and Material Efficiency, have wide applicability; others have key niche roles that are Industry-Specific; the Power Grid pillar requires interaction between grid decarbonization and industry action to switch from fossil fuels to zero-carbon electricity where appropriate.

## 1. Introduction

Industry is a major source of CO_2_ emissions in the United States (U. S.) and around the world. Absent large negative emission sources, mitigation of climate change will require large reductions from industry. This paper reviews a broad literature on technologies and other changes to industry that can reduce emissions. We summarize those actions and create a bottom up accounting exercise to access how much the U.S. industrial sector can achieve in emission reductions by 2050. The next sections expands the background of our study and provides more details on the methodology. The literature review covers the main industries included in the study. Finally, the estimates are presented and discussed.

Climate change, air quality, and other forms of pollution present challenges, especially to government and business as they seek solutions to reduce emissions of greenhouse gases to mitigate the effects of climate change. Simultaneously, long-term strategic issues make planning for the future important. Matters of significance include industries’ access to resources, governments’ interest in managing resources more efficiently and societies’ building sustainable economies into the future.

**Fig. 1.**
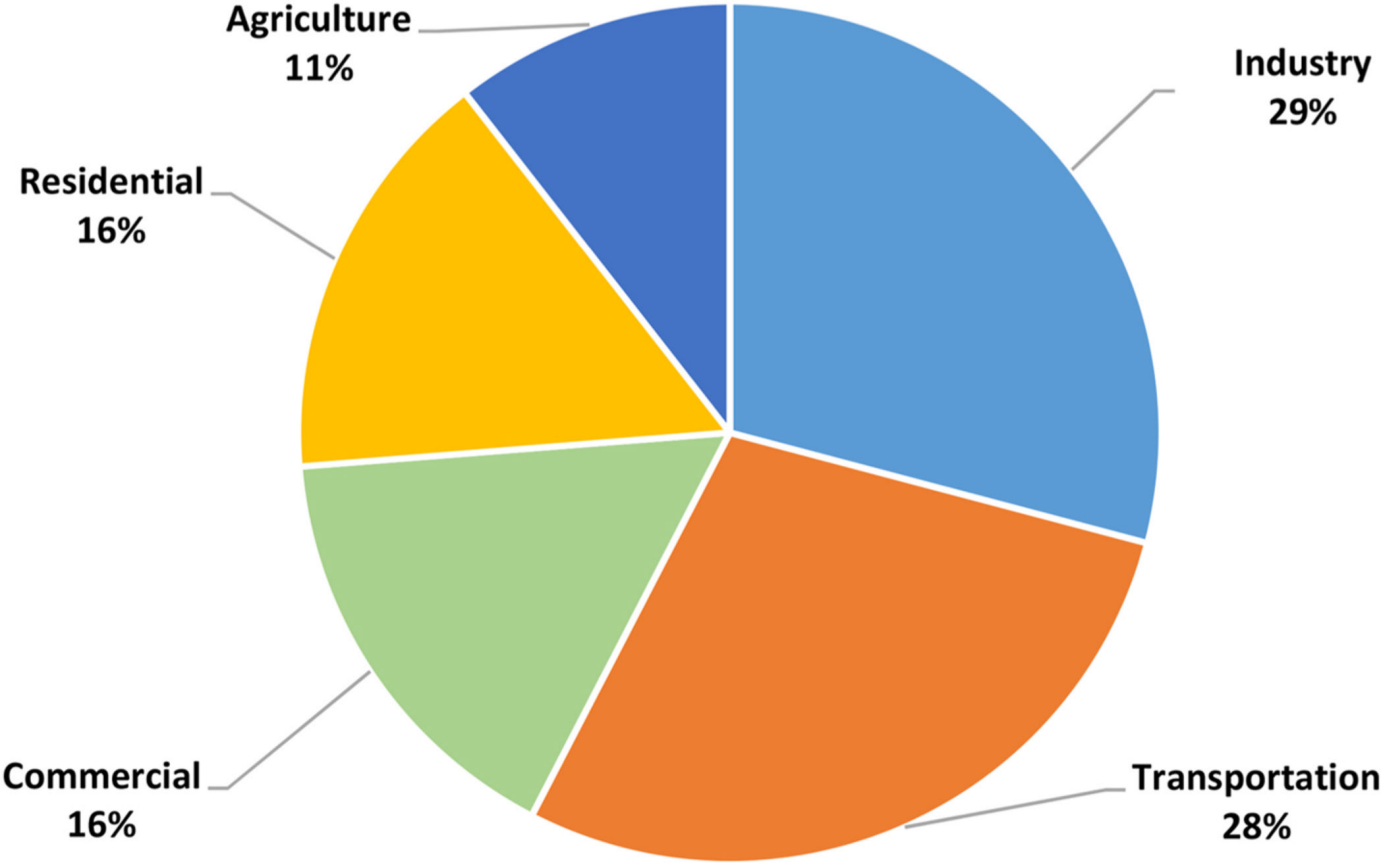
2018 energy related greenhouse gas emissions in the United States, by economic sector, with emissions from electricity production allocated to the sector (source: (U.S. Environmental Protection Agency, 2020)).

Manufacturing is the foundation of the global economy; it converts raw materials to products that are central to everyday life. Worldwide, industry is a significant source of greenhouse gas emissions and air pollutants, since the conversion of raw materials currently consumes large amounts of fossil energy. In the United States, industry is the largest source of energy related carbon dioxide (CO_2_) emissions (see Fig. 1).

This study examines the broad issues that underlie deep decarbonization of manufacturing (DDM) in the United States, with the primary emphasis on *quantifying the potential for energy-related CO*_*2*_ *emissions in 205*0. Unlike recent studies that provide a wealth of information and perspectives regarding various technologies and policies that could be used for deep decarbonization, we review those studies to synthesize a set of transparent quantitative assumptions on the potential contribution from four “pillars”, i.e., groups of actions that provide a plausible integrated approach to DDM. The pillars are *Energy Efficiency, Material Efficiency, Industry Specific* (e.g. hydrogen, Carbon Capture Utilization and Storage), and *Grid Decarbonization*. Assumptions derived from these other studies allow this study to quantify deep decarbonization in the year 2050. These estimates are not the result of modeling specific policies or price projections to achieve deep decarbonization, but instead are an accounting exercise designed to illustrate and quantify how the pillars might plausibly work together and to evaluate their potential contributions to support DDM. Throughout this paper, the notion of “feasibility” is used. We derive the estimates in this accounting exercise based on the assessments of other published studies that those individual actions described here are “feasible,” i.e. technically possible and economically attractive under policies to address climate change. This analysis is the cumulative impact of these actions. If any of those actions, described in these other studies, do not meet those technical and economic criteria then the cumulative emission reductions presented here will be lower. Note that if the social costs of climate change (see e.g. USGCRP, 2018) are included, it may make all opportunities economically attractive.

Globally, the industrial sector is responsible for 33% of greenhouse gas (GHG) emissions, including direct, indirect, and process emissions (Rissman et al., 2020); in the United States, the share of emissions is 29%. While industry is a major source of non-CO_2_ GHG emissions, CO_2_ emissions are the major share of GHG emissions, and energy-related CO_2_ emissions are the largest component of total CO_2_. To limit warming to the Paris Agreement target of 1.5 °C, emissions must reach net zero^1^ around 2050 (Intergovernmental Panel on Climate Change, 2018). Since industrial activity is both the core of the global economy and a major contributor to GHG emissions, the net-zero target requires similar reductions of industrial GHG emissions generally, and in particular of the energy related CO_2_ emissions, which is the focus of this paper.

U.S. manufacturing has had a long, successful history of improving both energy and associated emission intensity. The U.S. Energy Information Administration (2019b) reports that from 1998 to 2018 (the year of the most recent published data) manufacturing energy intensity declined by 26%; an annualized rate of − 1.5%. Not all energy intensity declines are efficiency improvements. Belzer (2014) estimates that from 1985 to 2007 delivered energy intensity declined at about the same rate, but that the changing composition of the U.S. industry accounted for about a third (−0.5%) of the decline in aggregate energy intensity. Since GDP has grown at an annual average rate of 1.9%, total energy demand and associated CO_2_ emissions have grown, but at a slower rate than economic activity. Lowering the energy and emission intensity of U.S. industry is a necessary, but not sufficient, condition to reach the Paris Agreement target of 1.5 °C. This paper explores how far additional structural changes to industrial material use in the economy, shifts to low- and zero-carbon energy sources, carbon capture, and other industry-specific technologies will take the industrial sector toward that goal.

Deep decarbonization of the U.S. economy in the context of a net-zero target has received attention from economy wide studies (e.g. Davis et al., 2018) and those that focus on the industrial sector. Several recent papers provide technical options for decarbonizing industry on a global level (Bataille et al., 2018; Rissman et al., 2020) or focused on the U.S. (Whitlock et al., 2020). Thiel and Stark (2021) highlight the importance of deep reductions of industrial emissions to achieve net zero-emissions. However, these papers do not quantify the role of specific technologies on the trajectory of future emissions. Other recent papers focus on specific technologies, e.g. hydrogen (Kazi et al., 2021), or industries, e.g. cement (Obrist et al., 2021) and steel (Wang et al., 2020). For U.S. decarbonization, (Larson et al., 2020), hereafter NZA, and (Sachs et al., 2020), hereafter ZCAP, are economy-wide studies that present scenarios that constrain GHG emissions in 2050 to net-zero. The industry specific sections of both NZA and ZCAP provide discussions of technical options for decarbonizing industry. Both raise the issue of the “hard to decarbonize” industrial sectors and discuss the options similar to this study.

Other recent country level studies of decarbonization examining China (Burandt et al., 2019; Khanna et al., 2019), use economy-wide integrated models to optimize emission reduction targets, but may lack industry specific technology detail. Other studies analyze specific energy or GHG policies for decarbonization. There are a wide variety of proposed policies to facilitate reduction of energy use and associated GHG emissions. DDM will have major policy implications for energy markets and the structure of the future economy.

This study takes a different approach. An optimization model sets a target, so the level of emissions (zero) is a foregone conclusion. This study looks at the individual technical options, or “pathways,” for decarbonizing specific industries and quantifies the emission reductions from the bottom up. If the individual options are *prima-facie feasible*, under a policy regime that prioritizes reducing impacts of climate change, then this bottom-up approach provides an estimate of how close to zero is *prima-facie feasible.* While different in approach to NZA, ZCAP, and other studies reviewed here, the goal is similar. This study adds to that evidence of *plausible pathways* to DDM in the U.S. There are a wide variety of policies proposed in the literature to facilitate reduction of energy use and associated carbon emissions. DDM will most likely be the result of a mix of policies to enable changes in industrial activity, energy, and emissions that result in a low-carbon emissions future.

Recognizing this, the study starts with the U.S. Energy Information Administration’s (EIA) Annual Energy Outlook (AEO) as a Reference Case forecast of industrial activity, energy use, and CO_2_ emissions (U.S. Energy Information Administration, 2019), hereafter the *Reference Case*. This study combines the forecast with a review of literature on industrial CO_2_ emissions technologies and practices to address the question: *“How much could industry reduce* CO_2_ *emissions by 2050?”* This study answers this question via a set of CO_2_ emissions-reduction estimates for U.S. manufacturing by 2050 based on a bottom up accounting of the U.S. manufacturing sector.

## 2. Methodology

This paper focuses on eight sectors; seven energy intensive manufacturing sectors and one aggregate “rest-of-manufacturing” sector. We conduct a bottom up accounting exercise to estimate emission reductions in each of the eight sectors and each of the four pillars for DDM. This section provides more detail on our approach.

**Fig. 2.**
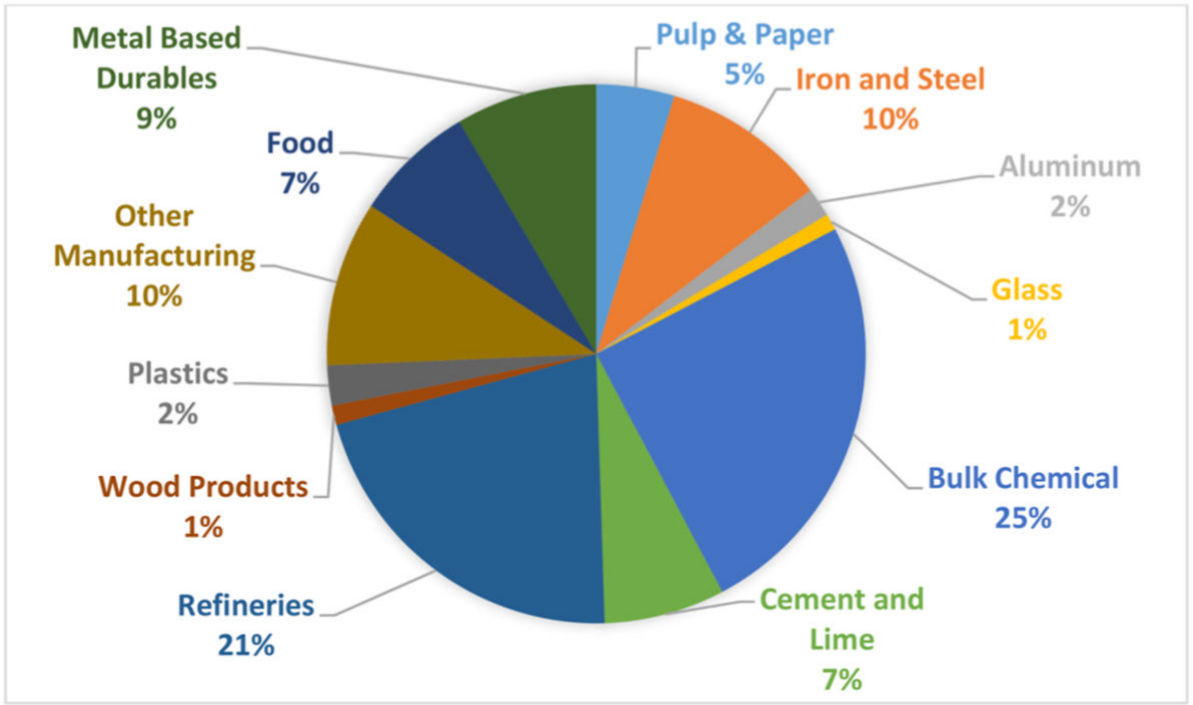
Energy-related manufacturing GHG emissions from key industries source: AEO 2019

Within the manufacturing sector in the United States, 71% of CO_2_ emissions are concentrated in the seven energy intensive industries; Pulp & Paper, Iron & Steel, Aluminum, Glass, Refineries, Bulk Chemicals, and Cement & Lime (see Fig. 2).

**Fig. 3.**
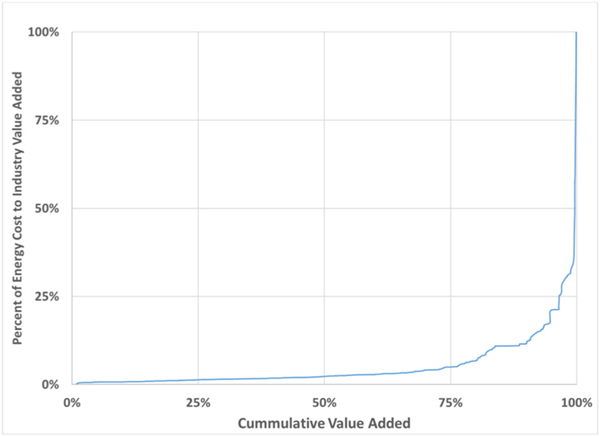
Percent of Energy Cost to Value Added by 6-digit NAICS Cumulative Value Added (Source: U.S. Census Bureau, 2016 and author’s calculations).

These industries are the most energy intensive when measured in terms of energy cost per dollar of value added (see Fig. 3). However, only 25% of total manufacturing value added comes from industries with energy cost share of value added greater than 5%, but these comprise 62% of total energy expenditures.

The nature of the highest CO_2_-emitting industries’ processes, with high-temperature and high-energy intensity, presents challenges to industry in the search for new technologies. In addition, the complexity of current industrial infrastructure and interlinkages slow change, e.g. challenges using waste energy or by product flows of one process by another process on the same site or at a neighboring plant.

For some industries (or parts of some of the more energy intensive industries), more benign operating conditions allow easier conversion to low-carbon processes or technologies; lower temperature steam in food processing or papermaking, for example. Yet, deep decarbonization of industry will require significant technological changes and innovation within a relatively short window of opportunity. In this study, we evaluate the cumulative potential of various technologies and material use changes to make deep reductions in CO_2_ emissions in U.S. industry by 2050. The study concentrates on seven of the most carbon- and energy-intensive industries: i.e. pulp and paper, iron and steel, bulk chemicals, cement and lime, oil refining, aluminum, and glass and a general assessment for the less energy-intensive portion of manufacturing, which we label “light industry,” in contrast to the energy-intensive, “heavy” industries. Light industry includes some modestly energy intensive sectors like food processing and metal casting but also a wide range of assembly and fabrication sectors that use primary materials produced by the up-stream heavy industry to create final consumer products.

Our approach uses a detailed set of DDM potentials from industry-specific actions as determined by a review of other studies and analyses (publicly available by early 2020). We convert the information from these studies into an estimate of the reduction in future emissions for the U.S. manufacturing industry, using a detailed accounting framework. It is important to be clear on what this framework encompasses. This study focuses on energy-related CO_2_ emissions from manufacturing, with the addition of process CO_2_ emissions from cement and lime manufacturing, which are the single largest component of industrial process CO_2_ emissions. We account for the specific circumstances of U.S. industry (e.g. structure, location) and technology characteristics. These estimates of potential reduction in energy and CO_2_ emissions are applied in a detailed accounting exercise relative to the Reference Case.

Quantifying DDM is a comparison of two alternative futures; a quantification of the outcome of individual CO_2_ emissions-reduction actions determined to be feasible in other published studies compared with one where no additional action is taken. The analysis builds upon a detailed set of assumptions regarding estimates of potential emission reductions and assuming that, since these potential emission reductions are feasible with the right incentives, there is a logical outcome in terms of CO_2_ emissions reductions. This analysis provides a quantification of DDM potentials, relative to the Reference Case via a set of transparent assumptions about actions that can feasibly reduce CO_2_ emissions. The study avoids double counting as much as possible, since some technologies may compete or overlap with respect to CO_2_ emission reductions.

The studies reviewed for this analysis provide evidence of the feasibility of reducing emissions in a variety of ways. The **four pillars** that support DDM include:

- **Pillar One—Energy Efficiency**: Energy efficiency offers large potential in the industrial sector. Technologies or strategies in this pillar include energy management driven by data analytics, additive manufacturing, and efficient process design/integration/. There is untapped potential for energy savings by implementing well-known technologies and practices such as combined heat and power (CHP), waste heat recovery, efficient motor systems, and energy management practices.
  - *Reference Case Efficiency:* Some energy efficiency is included in the Reference Case. To avoid double counting, the Reference Case efficiency is calculated by comparing the 2050 Reference Case energy use to a counter-factual, calculated by multiplying the forecast 2050 industry-level production by the industry-level energy intensity in the base year of the Reference Case. The difference between this “frozen efficiency” energy use and the forecast energy use is ***Reference Case efficiency***. The sum of Reference case emissions and emissions that would have arisen in the absence of Reference Case Efficiency is ***Potential emissions.***
  - *Incremental Efficiency:* This pillar considers potential efficiency improvements beyond the Reference Case based on other studies. When other studies show that higher rates of efficiency are feasible, the difference between the efficiency taken from the studies and efficiency included in the reference case is Incremental Efficiency.
- **Pillar Two—Material Efficiency**: Industry faces pressure as it seeks to access a finite amount of critical raw materials. Recycling and related measures could alter how industry uses raw materials and energy. In a “circular economy,” products and materials that are reused and recycled retain the highest value for the economy, leading to reduced production of energy intensive primary materials in favor of less energy intensive products. The largest impact will be for the material production industries but also in fabrication, if society moves from product ownership to shared-use products, e.g. automobiles.
  - *Refineries:* While we do not model the transportation sector, it is likely that factors driving DDM will affect energy demand in other sectors, in particular gasoline. This is represented as a decline in forecast refinery product output and lower associated emissions.
  - *All other manufacturing:* The changes described above imply that the output of specific material producing sectors will grow more slowly and associated energy use will decline with shifts to recycled feedstocks. This study captures these effects by lower forecast growth in output from specific manufacturing sectors.
- **Pillar Three—Industry-Specific:** There are several DDM opportunities that are likely to be effective in specific industries, including:
  - *Direct Use of Renewables*: Renewables may offer potential for direct on-site use at manufacturing plants.^2^ Direct on-site renewable energy uses include solar energy for drying or heat generation and the use of self-generated biomass residues as fuel, which are unique to some sectors.
  - *Hydrogen or renewable gases*: Hydrogen or other generated renewable gases (e.g., methane from renewable sources) in energy-intensive industries can replace fossil fuels. This offers the potential for deep reductions for gases produced at net-zero CO_2_ emissions. Low- or zero-carbon hydrogen can be produced through the combination of natural gas steam reforming with carbon capture and storage (“blue hydrogen”) or through electrolysis of water using renewable power sources (“green hydrogen”). If hydrogen production relies on a route from natural gas, consumption of blue hydrogen would be associated with emissions of methane or CO_2_ in the supply chain. For simplicity, this study assumes that green hydrogen is used.
  - *Carbon capture, Utilization & Storage and utilization (CCUS)* offer the opportunity to convert fossil fuels to low-carbon energy carriers or reduce energy- and process-related emissions in specific industries such as chemicals and cement. The captured CO_2_ can be stored underground (e.g., in depleted oil or gas reservoirs or aquifers) or can be recycled as fossil carbon into feedstocks or chemical products. CCUS can also contribute to deep emission reductions if the captured carbon is stored in products outside the atmosphere long term, and/or captured at the product’s end of life.
- **Pillar Four—Power Grid**: Decarbonizing power production in combination with electrification of manufacturing operations can offer much for DDM. This pillar estimates the impact on emissions from switching electricity demand to zero carbon sources and switching fuel use to electricity from zero carbon sources. Note that the decrease in energy demand is an important enabler of this pillar in 2050. In the absence of lower electricity demand and fuel demand, which could switch to electricity, the investment in grid infrastructure would need to be much higher to support this pillar.
  - *Zero CO*_*2*_ *Grid:* The manufacturing sector may actively participate in enabling grid decarbonization via on-site renewable generation or power procurement practices. The role of manufacturing companies to assist grid decarbonization goes further by providing load balancing. This study assumes that any electricity demand not reduced by energy and material efficiency is met from zero-carbon sources.
  - *Electrification:* With a zero-carbon grid, there are further opportunities for decarbonization through substitution of electricity for fuel. Generic electrification opportunities include electric boilers and heaters. Energy-intensive industries will require specific technologies, e.g. electric arc furnaces and membrane separation. Some electrification opportunities, e.g. heat pumps, mechanical vapor recompression, offer energy savings and may be cost effective in current markets (Mai et al., 2018; Rehfeldt et al., 2020). This study captures this by estimating how much remaining fossil fuel can switch to electricity, assuming that the electricity is from zero-carbon sources.

This study groups the four pillars into two categories, *Direct action* and *Interaction*. *Direct action* is composed of the first three pillars and is the result of actions taken within industry. The second estimate is the indirect emission reductions in the fourth pillar that arise from the *Interaction* of industrial electric power consumption, complemented by beneficial electrification, from zero-carbon sources. In other words, industry decarbonizes when it uses grid provided electricity and replaces fuel-powered equipment with electrified equipment both from zero carbon sources. Combining *Direct action* and *Interaction* represents the total emission reductions that comprise this study’s results.

This study starts with the AEO Reference Case, assuming current laws and regulations affecting the energy sector, are unchanged throughout a projection. The projections assume trend improvement in known technologies, along with other economic and demographic trends. While the AEO is an annual forecast through 2050, this study uses the AEO base year, 2018, and the terminal year, 2050; this study does not assess the timing of DDM actions. During the 30-year forecast horizon, we assume a 100% turnover of the energy-using capital stock based on currently available technology. In the AEO reference case, the rate of retirement in the National Energy Modelling System is 1.2% per annum for light industry, 1.7% for Chemicals, and straight-line retirement over 20 years for the other industries. Our assumptions overstate retirement in light industry and chemicals and understate retirement for the rest of heavy industry. All the results presented in this study indicate whether the estimates are relative to Reference Case or Potential Emissions, the latter of which excludes emissions from Reference Case Efficiency.

## 3. Industry-specific analysis

Table 1 provides the industry specific assumptions on the key technology and potential emission reductions derived from the literature. While new technology may become available between now and 2050, this study does not include this. Commercialization of industrial technologies may take decade. To the best of our ability, care has been taken to align the industry-specific studies with what is already embodied in the Reference Case, particularly for energy efficiency.

### 3.1. Pulp and paper industry

With a production of nearly 72 million metric tons (Mt) in 2018, the U.S. pulp and paper industry is the world’s second largest (FAOStat, 2021). The industry includes both integrated pulp and paper mills and stand-alone paper mills. The former produce virgin paper from wood, while the stand-alone mills primarily process recycled fiber and purchased “market” pulp. The large use of forest products makes the industry unique in the large volume of biomass used as both raw material and an energy source.

**Energy efficiency** is an important opportunity in this industry, as many mills use relatively older and small-capacity equipment. If all pulp and paper mills would move to the energy efficiency level of the top 25%, the industry could reduce energy use by 17% (Boyd, 2016). Miller et al. (2015) estimated primary energy savings of 45%–61% if the industry would completely shift to best-available technologies. These potentials include more efficient drying technologies, increased heat recovery, increased use of CHP, as well as increases in efficiency of motor systems (e.g., mill drives, pumps, and fans). Miller et al. (2015) estimated that improvements of up to 20% over current best available technology (BAT) would be feasible with technologies under development or in research, e.g. new bio-based pulping technologies as well as new electric-drying technologies. The European paper industry assumes additional energy savings of advanced technologies under development at 15% (Confederation of European Paper Industries, 2017). In this study, we assume fuel savings of 24% and electricity savings of 16% against the reference case.

**Material efficiency** is primarily driven by the packaging market, as two-thirds of annual production is sold into this market, with printing papers a good second. Packaging demand is coupled to the state of the economy and population (Rouw and Worrell, 2011), and has benefited from the trend toward increased internet sales. In the Reference Case, a 1% annual growth of paper production is forecast. Increased pressure on single-use plastics is expected to positively affect paper use. We do not expect a decline in consumption relative to the baseline. Corrugated board can be produced from 100% recycled fiber. This allows for an increased share of recycled fiber in the industry. While not all waste paper is recovered in the United States, the country also exports about 18 Mt of waste paper. In line with a growing interest in the move toward a circular economy, we assume that waste paper use will increase. We assume that the use of recycled fiber will increase from today’s 37%–56% by 2050, consuming all currently available recycled fiber in the United States. This will reduce demand for pulp and result in energy savings of approximately 320 PJ, or approximately 15% of total energy demand.

#### Renewables.

The pulp and paper industry is already unique in its reliance on a high share of self-generated biofuels in the integrated chemical pulping plants. Use of renewables in integrated plants can be increased by better use of harvesting residues. New technologies like black liquor gasification could increase the generation of power, compared to the current steam-turbine systems. This technology has been under development for a long time and has yet to make a break-through. In addition, based on potentials for the Swedish pulp and paper industry (Ekstrand et al., 2013), we estimate that 1% of the total heat demand of the industry could be replaced by self-generated biogas through anaerobic digestion of waste water. In Europe, the potential for deep geothermal energy for paper mills is being explored. No studies have examined the potential for geothermal energy in the U.S. pulp and paper industry. Use will depend on the location and local geothermal conditions.

#### Hydrogen.

There is limited potential for the application of hydrogen in the pulp and paper industries. It can be used to replace current use of natural gas as fuel in CHP units and boilers, but the use of hydrogen does not offer any synergies or other additional benefits and, hence, may be expensive.

#### CCUS.

Theoretically, the use of CCUS in the pulp industries would allow for the realization of so-called negative emissions, as CO_2_ absorbed by the biomass would be stored underground. In contrast to most other studies and roadmaps, the IEA global ETP scenarios assume CCUS is applied to 40%–50% of new pulp and paper mills (International Energy Agency [IEA], 2012). However, given the typical size and location of pulping facilities, adding CCUS facilities at U.S. pulp mills seems unrealistic, certainly in the time frame to 2050.

#### Electrification.

The industry has tested various electric drying technologies in the past decades, but they have made little impact due to the relative high cost of electricity compared to steam. The industry can use Infrared (IR) drying, with novel electric drying technologies using IR or microwave technology under development (Schüwer and Schneider, 2018). Other opportunities include the use of electric boilers to generate steam for the paper machine and direct electric heating of cylinders may reduce the heat losses in steam production. Heat pumps and transformers can produce steam from the waste heat recovered from the closed hood of the paper machine (ECN, 2013). No studies have estimated the full potential of electrification in U.S. paper mills (Jadun et al., 2017). A study for the UK paper industry estimated that electrification can contribute 22% of a full reduction of fossil energy demand in the industry (WSP Group & DNV-GL, 2015).

In the long term, the pulp and paper industry will be one of the industries with considerable renewable-based CHP capacity. While heat demand is almost continuous in modern pulp and paper mills, the use of back-pressure turbines and heat storage may offer this industry the opportunity to offer load-balancing services by offering dispatchable power supply.

### 3.2. Iron and steel industry

Primary steel is produced from iron ore, while secondary steel is made from recycled scrap steel. In 2017, the U.S. steel industry produced nearly 82 Mt of steel, of which 67% was produced in Electric Arc Furnaces (EAF), using primarily scrap, while 33% came from primary steel plants. Iron production was 22 Mt of pig iron and 2 Mt of Direct Reduced Iron (DRI) (Worldsteel, 2018).

**Energy efficiency** is a key enabler of the transition to a sustainable steel industry. Jamison et al. (2015) estimate the potential of energy efficiency improvement in the U.S. steel production by switching to best available technologies at 39%. Improvements are mainly located in the basic oxygen furnace and hot rolling mill. New technology could result in additional energy savings (estimated at 24% by Jamison et al., 2015). These figures are roughly in line with other estimates (Hausker et al., 2015; IEA, 2012). In this study, we assume fuel savings of 11% over the reference case, as well as electricity savings of 14%.

New technologies in ironmaking are being developed that are more energy efficient – for example, smelt reduction using the Hisarna and Finex processes, as well as near-net-shape casting to replace casting and rolling. The choice of future ironmaking technology in a climate-constrained world will depend on local factors, such as the availability of low-cost hydrogen or storage options for CO_2_.

**Material efficiency** is mainly determined by the needs of the building, construction, and automotive markets. While the use of scrap is already high in the United States, Pauliuk et al. (2013) expect that scrap availability will increase in North America. We optimistically estimate that the share of scrap use in EAFs could increase to 80% of steel production by 2050 (Cooper et al., 2020), and iron production would decline to 16 Mt/year.

#### Renewables.

Due to the high temperature nature of steelmaking processes, any form of energy needs to be highly concentrated. This limits the direct use of renewables like biomass. Biogas and synthetic methane (from biomass) could be used as a fuel for reheating furnaces or injection fuel in the blast furnace. For this analysis, we assume that the share of renewables in the steel industry will be very limited.

#### Hydrogen.

Today, two alternatives for ironmaking are the key candidates to replace the blast furnace in ironmaking: smelt reduction, such as the Hisarna process, or reduction using hydrogen. ThyssenKrupp is piloting the injection of hydrogen to replace coal. SSAB is involved in a pilot project in Sweden, the HYBRIT-project. Electrolytically produced hydrogen (using hydropower) will be used to reduce iron in a DRI hot briquetted iron (HBI) plant. The HBI will be used as iron input in an EAF (Vogl et al., 2018). Vogl et al. (2018) estimate the electrical power consumption of this route at 3.5 MWh/mt of steel.

#### CCUS.

CCUS is only an option for large CO_2_ sources, which are found in integrated iron and steel mills. As blast furnace gas has a high concentration of CO_2_ (and CO), the ironmaking is a good candidate for CCUS. A pilot project to develop a circulating blast furnace to test capturing CO_2_ in ironmaking in France was cancelled, so practical feasibility remains unknown. Since CCUS will require additional inputs of energy to process large volumes of BF gas, smelt reduction processes may be a better candidate for CCUS, which would result in lower costs for CCUS (Keys et al., 2019). For this study, we assume that a combination of smelt reduction and CCUS is the most viable option for sites that have access to CO_2_ storage. The Hisarna process produces energy savings of 20% compared to the state-of-the-art ironmaking in blast furnaces and has a smaller footprint and capital costs than current technology (van der Stel et al., 2013; Gerres et al., 2019). The process allows for the increase of scrap additions, increasing the opportunities to recycle low-quality scrap.

#### Electrification.

The shift toward more production in EAFs will increase the use of electricity. Electrification of other furnaces may be feasible using induction or plasma furnaces. Electrolytic reduction of iron oxide is both researched in Europe and the United States (Fischedick et al., 2014), but is not expected to be commercially available before 2040 (Eurofer, 2013; Quader et al., 2015). Hence, for this evaluation, electrification of the steel industry is assumed to be driven by the shift towards more production in EAFs and electric (induction or plasma) furnaces.

### 3.3. Bulk chemical industry

The bulk chemical industry produces an enormous variety of products used in almost all parts of our lives. However, energy use is concentrated in a few “platform” chemicals that form the basis for an array of chemical products. These are the petrochemical building blocks (e.g., ethylene, propylene, butadiene), ammonia to produce nitrogenous fertilizers, chlorine and alkaline, methanol, and industrial gases.

The U.S. chemical industry is the largest fossil fuel consumer, with an estimated consumption of nearly 6.8 EJ (2014), of which about 4.0 EJ is used as feedstock (2018; U.S. EIA, 2021). With 147 TWh, it is also the largest industrial power consumer. The industry is the largest user of CHP.

#### Energy efficiency.

The chemical industry is a highly integrated industry with facilities that operate multiple process units to produce a variety of products. Integration is a major source of energy efficiency improvements. Many energy savings opportunities focus on more efficient use of heat through better process design of reactors and distillation/separation processes (e.g., advanced distillation or membranes), or through improved heat recovery within processes and through process integration. This may include technologies that accelerate electrification of the industry (e.g., heat pumps, mechanical vapor recompression, membranes). Brueske et al. (2015a) estimate potential energy savings of 19% with currently available technology in the U.S. chemical industry, where the savings in ammonia production are higher than in olefin production. Advanced technology could add another 31%. Other studies estimate a potential savings of between 13% and 35% (Saygin et al., 2011; IEA, 2012), with varying assumptions on included processes (e.g. Yao et al., 2015 focuses solely on ethylene production), technology availability, and geography (Hausker et al., 2015; CEFIC, 2013). In this study, we assume additional 18% fuel savings and 14% electricity savings over the savings already included in the reference case.

#### Material efficiency.

While demand, and especially exports, of the U. S. chemicals industry have grown recently, studies that focus on decarbonization expect that chemicals demand will decline. This may be due to the transition to a circular economy and increased material efficiency, including actions to reduce plastic littering, bans on single-use plastic products, and reduction of fertilizer use coupled with increased efficiency in agricultural practices. Of all materials in society, plastics are recycled the least, e.g. the U.S. recycling rate is about 9% (Geyer et al., 2017). Recycling rates are expected to increase globally.

Few older studies of deep decarbonization of the chemicals industry include demand-side measures, and the newer ones that do include demand-side measures show a wide variation in the extent that it contributes to decarbonization. Estimates of the reduction in the production of virgin plastics through increased material efficiency in product design and recycling (Bazzanella and Ausfelder, 2017; Elser and Ulbrich, 2017) varies widely, from 7% to 55% (IEA, 2018; Stork et al., 2018). Plastic recycling decreases the energy used to make a ton of plastic by 25%–55%, depending on the recycling process and plastic. Estimates of the reduction in fertilizer use also vary widely, between 20% and 50% (IEA, 2018).

#### Renewables.

Renewable feedstocks (i.e., biomass) have been used for the manufacture of some bulk chemicals. Bioplastics (Shen et al., 2010) are available commercially but still represent only a fraction of the total plastics market. Developments focus on the production of new platform chemicals (especially for oxygenated chemicals), while others focus on replacing current fossil-based chemicals (e.g., ethylene, butadiene, caprolactam and others). The potential contribution of biomass feedstocks in the chemical industry varies in the studies between 5% and 20% of petrochemical feedstocks and methanol by 2050 (IEA, 2018; Griffin et al., 2018). A study for the Dutch chemical industry assumes a 35% reduction of CO_2_ emissions (including feedstocks) by shifting to biomass-based feedstocks (Stork et al., 2018).

Other forms of renewable energy may also be used in the industry to provide heat or power (e.g., deep geothermal energy to supply steam or direct use of solar energy). Application of renewables will depend on the processes used and location.

#### Hydrogen/biogas.

Hydrogen, biomethane or liquid bio-based fuels are an option to replace fossil feedstocks or as a fuel in high-temperature furnaces. Some of the self-produced gases in the industry contain hydrogen. While CO_2_-free hydrogen can be produced by electrolysis from water or from natural gas using CCUS, this second route for hydrogen receives special attention in the context of carbon capture and *utilization* (CCU), to upgrade captured fossil-based CO_2_ to produce a chemical product. As long as the product does not decompose or is not incinerated, the CO_2_ will be stored in the product. Producing CCU-based fuels or single-use plastics will only delay emissions to the atmosphere and may not result in significant emission reductions. IEA (2018) assumes emission reductions of 10% using electrolytic hydrogen for ammonia, methanol, and CCU applications.

Biogas may be produced as biomethane (from anaerobic digestion of wet biomass) or syngas (from thermal gasification of biomass). New processes to directly produce hydrogen through biological processes or direct solar generation are under investigation but are not close to commercial application. Research programs around the world are looking at reducing the costs of electrolytic hydrogen production (e.g., using PEM fuel cell technology and other approaches). The energy efficiency of current commercial electrolytic production is around 60%–70% and could improve to 70%–80% (Carmo et al., 2013; Kumar and Himabindu, 2019). Hence, the use of hydrogen may lead to increased demand for (renewable) energy. Actual application will strongly depend on the opportunities to produce hydrogen cost-effectively. Few studies have estimated the potential for hydrogen in the chemical industry by 2050. The IEA estimates that hydrogen could reduce CO_2_ emissions in the chemical industry by 10% in 2050 (IEA, 2018).

#### CCUS.

CCUS offers an opportunity for emission reduction in the chemical industry. Ammonia, hydrogen and ethylene oxide plants already emit an easily be captured pure CO_2_ stream. Other processes would need to be adapted for the use of CCUS, with a focus on large furnaces. A special option is a centralized hydrogen unit in combination with CCUS with hydrogen as on-site fuel. Various studies estimate the role of CCUS to contribute to an emission reduction of 17%–20% of emissions (IEA, 2018; Boulamanti and Moya, 2017). No specific estimates exist for the U.S. chemical industry.

#### Electrification.

In recent years, there is a growing interest in electrification as an option to reduce direct CO_2_ emissions. Some of the energy efficiency options shift energy use from heat to electricity and are included in electrification. As electricity supply is becoming less carbon intensive, electric technologies become more interesting. Various companies (e.g. BASF, Linde, Sabic) explore electric heaters of ethylene cracking. Electric boilers are already used in the industry. New electrochemical processes are being specifically developed; for example, electro-catalysis to produce formic acid, ethylene glycol, iso-propanol, and acetic acid (van Kranenburg et al., 2016). For the key bulk chemicals, no electric processes exist in the literature. A small number of studies have estimated the potential for electrification in the chemical industry in the long term. A study for the Dutch chemical industry estimates a 7% reduction in CO_2_ emissions by 2050 (Stork et al., 2018). An analysis of the potential of electrification estimates that 20%–25% of current fuel use could be replaced by electric heating (van Kranenburg et al., 2016) in processes with temperatures over 200 °C.

### 3.4. Cement and lime industry

In 2018, U.S. cement production was estimated at 86.4 Mt (USGS, 2020a), making the U.S. the largest cement manufacturer after China and India. Cement is made from clinker and additives. In making clinker, limestone is calcinated, resulting in process CO_2_ emissions. About half of the emissions from the U.S. cement industry are from the calcination reaction. The average clinker-to-cement ratio in the United States is approximately 0.9. Clinker production consumes virtually all the fuel in the cement industry; raw material preparation and finish grinding consume most of the power. The latest energy data (2015) estimates electricity use at 11.4 TWh (or 138 kWh/t cement) and fuel use at 338 PJ (or 4.0 GJ/t cement) (USGS, 2017).

#### Energy efficiency.

Further reductions in energy demand are technically feasible. Modern cement kilns use pre-calciner and multi-stage preheaters to use energy efficiently. Modern roller presses would save significant amounts of electricity. However, few breakthrough technologies exist that would lower energy use further. In 2015, the U.S. cement industry consumed 4.4 GJ/t of clinker and 138 kWh/t of cement (USGS, 2017). Best practice technology would consume 2.8 GJ/t clinker and 62 kWh/t cement (Worrell et al., 2008b). Assuming all plants in 2050 would operate at the current best practice level, fuel efficiency could improve by 35% and electricity use by up to 55%. Schwartz et al. (2017) estimate the potential for energy efficiency improvement with current technology at 34%, with a further 4% potential with technologies currently under development. We assume a 34% reduction in fuel and electricity use by 2050 is feasible.

#### Material efficiency.

Cement demand is driven by growing population and affluence, and typically declines as society develops. This is in the reference case. Cement demand can be reduced by improving building design, reduction of concrete wastage at the construction site, as well as recycling of concrete. Based on various studies (Favier et al., 2018; Scrivener et al., 2018), we assume that cement demand can be reduced by 15% compared to the Reference Case.

A portion of clinker can be replaced by adding alternative binders to cement, such as fly ash, blast furnace slags, and natural pozzolan materials. Current ASTM standards in the United States allow up to 5% limestone addition. However, blended cements using significantly larger shares of slags, fly ash or limestone are uncommon in the U.S., in contrast to Europe, China, and other countries (Bhatty et al., 2011). For deep decarbonization, we assume that the U.S. also would move to increased acceptance of blended cement. In line with global IEA scenarios (IEA, 2012), we assume that the clinker-to-cement ratio can be reduced to 0.7, from 0.9 now. Note that reduced iron production and use of coal in power generation will ultimately result in reduced production of additives, although fly ashes and slags produced today and not used efficiently may be available for future use.

#### Renewables.

Except for on-site power generation, replacing fuels in the kiln with biomass is a key option to increase the use of renewables in the cement industry. Currently, many plants use alternative fuels (e.g., tires, refuse-derived fuels, chemical wastes), which may still contain fossil carbon. Switching to alternative fuels that contain biogenic material offers an opportunity to offset fossil fuel-based emissions. The type and design of the kiln, as well as the moisture content of the fuel, will determine the maximum use of biomass. Based on various studies (IEA, 2012; IEA/WBCSD, 2018), we estimate that on average 30%–50% of the fuels could be replaced by biomass. For this study, we assume that U.S. plants could shift to 30% biomass fuels. Further increases might be possible by pre-treating (e.g., torrefaction) or gasifying (either by anaerobic digestion or thermal gasification) the biomass, allowing for favorable combustion temperatures in the kiln.

#### Hydrogen.

Hydrogen can be used as a fuel in clinker kilns, but adaption of the burner and kiln design may be needed for good heat transfer to the material. As far as the authors are aware, this option has only been analyzed in studies, and no R&D is currently ongoing.

#### CCUS.

Alternative raw materials are being investigated, as well as alternative cements (e.g., geo-polymers and magnesium-based cements). However, limestone’s abundance makes it a low-cost raw material for cement making for the near future. Because of the process emissions, the cement industry would be a good candidate for CCUS. Various systems have been studied and tested e.g., post-combustion capture and calcium-looping. CO_2_ recovery from a calcium-looping system would exceed 90% (Schakel et al., 2018), including process emissions. CCUS would lead to a significant increase in energy use in the plant, even doubling energy use in some cases (IEA, 2012). Also, the CO_2_ needs to be stored, and not all cement plants are located near potential storage sites. An alternative may be CO_2_ curing of concrete (instead of using water) or other uses of the CO_2_. CO_2_ curing may reduce emissions by up to 300 kg/t cement for special cements (Favier et al., 2018; IEA, 2012). Various technologies claim to do this e.g., Solidia (Scrivener et al., 2018), while various cement companies are involved in these developments e.g., CEMEX, LafargeHolcim, and Heidelberg.

#### Electrification.

Theoretically, limestone could be heated with electricity, but at current prices this is economically unattractive. No electric clinker plants currently exist, but the opportunity is being studied for a cement plant in Sweden. In the CemZero-project, Cementa AB and Vattenfall are investigating the feasibility of changing the 2.5 Mt/year Gotland plant to an electric process (Edwards, 2021). It is too early to evaluate the feasibility of this application for the United States.

### 3.5. Petroleum refining industry

Petroleum refineries convert crude oil to a variety of products: fuels, petrochemical feedstocks, lubricants, asphalt, and other products. Over 70% of the refinery output consists of fuels, with the remainder being mainly petrochemical feedstocks and residues. Energy consumption in a refinery is a function of the type of crude oil processed, the mix of products produced, and the efficiency of the refinery processes. In 2018, U.S. refineries consumed 3.0 EJ in fuels and purchased 49 TWh of electricity (US EIA, 2021). Of the fuels consumed, 2.1 EJ are self-generated fuels, with the remainder being mainly natural gas. The industry is a large user of CHP, after chemicals and pulp and paper.

#### Energy efficiency.

Opportunities for energy efficiency improvement exist in petroleum refining. The bandwidth study for the U.S. DOE finds a 14% efficiency improvement with current technology, and 26% additional energy savings with technologies that are in various stages of R&D (Brueske et al., 2015b). Note that part of this potential is included in the Reference Case estimates. Also, future restructuring of the industry may reduce this potential, as it might be expected that smaller and less efficient refineries would be closed first.

#### Material efficiency.

In most deep decarbonization scenarios, the transportation sector is undergoing a dramatic change by moving to electric forms of mobility for passenger transport. The scenarios vary with respect to heavy-duty vehicles, aviation, and shipping. Jet fuel represents about 15% of current refinery output, while feedstocks and similar products represent another 15% of refinery output. Biofuels may play an important role in these sectors. The plastics industry is investigating biomass-based alternatives, while it is expected that increased material efficiency may reduce demand for plastics and, hence, chemical feedstocks.

Therefore, the petroleum refining industry of the future may be much smaller and have a very different structure. New refineries under construction in the Persian Gulf region and China already are designed to produce up to 50% feedstocks. The future of the U.S. refinery industry is difficult to forecast, as it may be driven by export markets. IEA assumes that global gasoline demand will decrease by 70% by 2050 (IEA, 2018), while a deep decarbonization scenario for California resulted in an 86% reduction of refinery output (Mahone et al., 2018).

#### Electrification.

As two-thirds of a refinery’s consumed fuels are self-generated, electrification is not attractive. Refineries that focus on chemical feedstocks will still generate fuels for self-consumption. Direct electrification as in other industries may therefore be of less importance for this industry. Electrification may take place as part of energy efficiency improvement through the introduction of heat pumps and mechanical vapor recompression for waste heat recovery, and the use of membranes to replace part of the distillation processes. We, therefore, do not assume an important role for electrification in this industry.

#### Hydrogen/biomethane.

Furnaces in a refinery can be fired with hydrogen and biomethane. One study assumed up to 30% use of biogas in the refining industry (Mahone et al., 2018), but this study did not use a refinery model to evaluate this option. Hydrogen is another option, when combined with centralized reforming of self-generated fuels and CCUS (see below).

#### CCUS.

CCUS is a key option for the petroleum refining industry. If oil products will still be used in a few sectors, petroleum refining remains necessary and will always produce carbon-containing, self-generated gases. This makes CCUS a necessity. CCUS can be applied to the main combustion sources: e.g., crude distillation unit, crackers, steam reformer, CHP/boiler. This will result in residual emissions from smaller furnaces. An alternative option is a centralized reformer to produce hydrogen for all combustion processes that is combined with CCUS. CCUS may result in emission reductions between 56% and 72% (WSP Group and DNV-GL, 2015; Morrow, 2018).

#### Renewables.

While refineries already mix in bioethanol to produce gasoline blends, and dedicated bio-diesel plants exist, it is not expected that refineries will use large volumes of renewable energy.

### 3.6. Aluminum and glass

The EIA Reference Scenario distinguishes two smaller energy-intensive industries: aluminum and glass. Historically, these were large energy consumers in the United States, but their share of industrial energy use has declined over time.

#### 3.6.1. Aluminum

The character of the aluminum industry has changed in the past decades with the closing of primary aluminum smelters. By 2018, the United States produced 4.6 Mt of aluminum, of which only 20% was primary aluminum. The remainder was produced from new (46%) or post-consumer scrap (34%) (USGS, 2020b).

##### Energy efficiency.

There is additional room for energy efficiency improvement in primary smelters (Kermeli et al., 2015). New technologies such as inert anodes and new cell designs have the opportunity to impact energy use in the industry. We expect that there is limited potential beyond the energy savings in the reference case, so we limit this to 10% beyond the Reference Case.

##### Material efficiency.

The United States remains an exporter of old scrap (1.7 Mt; USGS, 2020b), indicating that there is additional potential for recycling. Where in the past, production of primary and secondary aluminum were separate markets, scrap now can be added in the smelter to the primary aluminum, up to 30%. Using part of the exported scrap domestically would allow primary smelter production to decrease by about 25%.

##### Renewables.

The remaining primary smelters are found in New York and the Ohio Valley. As the share of renewables in the power mix increases, the associated CO_2_ emissions will decline. Remaining CO_2_ emissions are found in production and consumption of anodes and the production of secondary aluminum.

##### Electrification.

For primary smelters, electricity is already the key energy source. The ongoing development of inert anodes is expected to eliminate the anode-related emissions, while also reducing the emissions of perfluorocarbons (PFCs). Furnaces used in secondary aluminum manufacturing, as well as holding and ingot furnaces in the primary smelters, could be converted to all electric, as are found currently in some casting facilities (Kermeli et al., 2016), or could be converted to use hydrogen instead of natural gas.

#### 3.6.2. Glass

Glass production in the United States is about 20 Mt, of which half is container glass and the remainder is comprised of flat, fiber, and specialty glass. Glass is manufactured in natural gas-fired furnaces where a mix of materials (silica, limestone/dolomite, soda ash) are melted at high temperatures. Full electric furnaces are used for specialty and fiber glass, while electric boosters are used in the larger furnaces to increase furnace capacity. The industry uses cullet, recycled waste glass, to replace some raw material, especially in container glass production.

##### Energy efficiency.

There is additional room for energy efficiency in glass making as new, efficient furnace technologies are developed (Worrell et al., 2008a; U.S. DOE, 2017). The additional potential is estimated at 33%, while technologies under development could add another 9% savings (U.S. DOE, 2017). The key opportunities for improving furnace efficiency are through better heat recovery, more efficient burners, and advanced future furnace designs that are more thermally efficient.

##### Material efficiency.

The recycling rate in the U.S. glass industry is lower than in e.g. Europe, demonstrating that there is additional potential for recycling. Recycling saves energy and process-related emissions in the furnace (Rue, 2018). It is estimated that about 11 Mt of glass waste is produced in the United States. Recycling 80% of container glass waste could potentially reduce emissions by 2 MtCO_2_. Next to recycling, material efficiency opportunities exist to reduce the weight of bottles. We assume an average 10% reduction in the weight of bottles. Note that flat glass consumption will likely increase due to the need for more efficient buildings.

##### Electrification.

Fully electric furnaces are already used by specialty glass makers. Research is ongoing to scale this technology to the larger furnaces found in the other segments of the industry. For example, a large scale container glass hybrid furnace (using 80% electricity) will be built in Germany in a collaboration of European glass manufacturers. We assume that full electrification of the furnaces is possible by 2050. While hydrogen could be used as fuel, we do not know of any research into hydrogen-fueled furnaces.

### 3.7. Light industry (non-energy-intensive industries)

Beyond the energy-intensive industries, the U.S. manufacturing sector consists of a diversity of industries. The total 2014 fuel use by these “light” industries was 2.43 EJ, with final electricity use equaling 1.69 EJ (EIA, 2018). The key energy consumers among these industries are the food and beverage, fabricated metals, and transportation equipment industries (U.S. EIA, 2021). Due to the wide variety, we do not discuss these industries individually; instead, we take a more generic approach based on the typical end-uses for fuel/heat and electricity. Fuel and heat demand can be subdivided based on the application (e.g., direct heat, steam, hot water) and temperature levels. Except for a few electrolytic processes (e.g., metals) and electric heating (e.g., casting), more than two-thirds of electricity is consumed in motor systems. These applications will determine the technological opportunities to reduce the CO2 emissions. Fuel is primarily used in boilers (31%) to produce hot water and low-to medium-pressure steam, as well as in process heating (44%), and space heating and cooling (17%). Electricity is used in motor systems (40%), facility heating ventilation and cooling (HVAC) and lighting (26%), and ovens and furnaces (13%).

#### Energy efficiency.

For a number of these light industries, Energy Guides produced for the U.S. Environmental Protection Agency’s ENERGY STAR program have identified a wide variety of energy efficiency measures that are currently commercially available.^3^ In motor systems, savings of around 30% or more can be achieved (see e.g. McKane and Hasanbeigi, 2011). Also, new technologies are under development that can further increase the potential savings. Some of the new technologies (e.g., heat pumps, mechanical vapor recompression) would also contribute to electrification (see below), making it difficult to decompose the reductions in CO_2_ emissions. Based on a variety of broader studies (e.g., Aas et al., 2018; Fais et al., 2016), the ENERGY STAR Energy Guides, and statistical data seen through manufacturing plant energy performance benchmarks developed for the ENERGY STAR program (for example, see Boyd, 2017), we estimate an energy efficiency improvement potential of 25%–30%. For this study, we assume savings of 25% for fuel end uses, and 30% for electric end uses (e.g., motor drives, HVAC, lighting).

#### Material efficiency.

Changes in final demand of the products made by these industries is difficult to estimate as the variety in products is so large. Yet, in a world view consistent with deep decarbonization, it can be expected that demand will decline relative to the assumed baseline. For example, current food waste in the food supply chain is estimated at 25–30% (Gustavsson et al., 2011; Kummu et al., 2012), and efforts are underway to reduce these losses. Additionally, new manufacturing technologies (e.g., additive manufacturing, Industry 4.0) are likely to reduce production losses. Uncertainties make it difficult to produce an ex-ante estimate of how these technologies will affect the losses. We assume that demand will be reduced by 10% (on average) across all other industries, relative to the reference case.

#### Renewables.

Depending on local conditions, parts of these industries could use renewable energy on-site to generate heat and power. The type of renewable energy, as well as the contribution it can deliver, are driven by the specific local situation. As potentials depend on local circumstances, those identified in various studies vary widely, from as low as 5% (only biomethane) to about 25% for all renewables, with an emphasis on biomass (e.g., Aas et al., 2018; White House, 2016). For this study, we assume that 25% of heat demand can be met by renewables. While CHP applications also will generate electricity, no studies have explicitly estimated the contribution for the United States. We assume that half of the heat is produced in biomass-fired CHP units (and some geothermal), with an assumed power-to-heat ratio of 0.25 (i.e., for every kWh of heat, 0.25 kWh of electricity is generated).

#### Hydrogen.

While there are some specific high-temperature processes in selected industries (e.g., furnaces in the non-ferrous metallurgical and metal processing/casting industries) in which hydrogen could potentially be beneficial, in most other industries hydrogen would be a less attractive fuel compared to the alternatives of electrification and renewables.

#### CCUS.

Most of the plants in these light industries would have relatively small volumes of emissions, making these industries less likely candidates for carbon capture and storage, as the costs for capture and infrastructure will be high. Industries that are co-located with larger industrial sites and emission sources might benefit from better CCUS economics, but in this analysis we do not assume that CCUS will play a discernible role in these industries.

#### Electrification.

Most end uses for low- or medium-temperature heating in these light industries are relatively easy to electrify. Electric boilers and heat pumps can generate hot water and low-pressure steam. Heat pumps and mechanical vapor recompression (MVR) for heat recovery and generation of low-pressure steam from waste heat with a coefficient of performance of 3 (i.e., for every unit of electricity, 3 units of heat are generated) are currently being demonstrated in various applications (Arpagaus et al., 2018), while small-scale electric boilers have been used for decades. Electric furnaces are used in virtually all industries, typically in smaller units. Upscaling of these technologies would be necessary. For this study we assume that, of the remaining hot water and steam demand, about 30% can be generated with electric boilers (replacing 1 unit of heat by 0.9 units of electricity) and 20% by heat recovery using heat pumps (replacing 1 unit of heat by 0.3 units of electricity). The remaining heat can be generated by on-site renewables (e.g., biomass waste flows, geothermal or solar thermal). We assume that all remaining process heat demand can be replaced by fully electric alternatives (replacing 1 unit of heat by 0.9 units of electricity).

## 4. Results and discussion

**Fig. 4.**
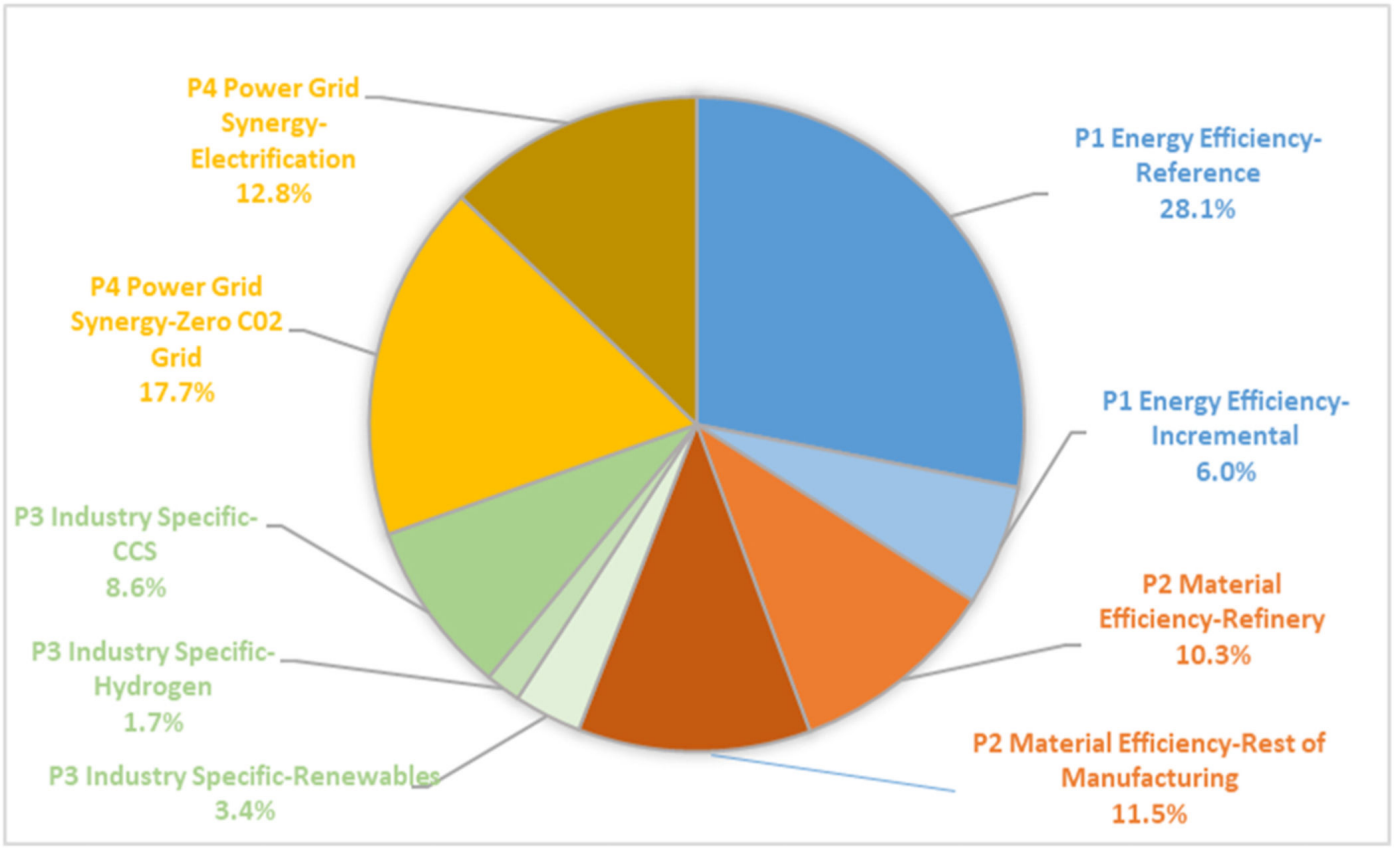
Relative contributions of the four pillars of DDM, with detailed components.

This study finds that implementing the actions described in Table 1 could reduce reference case carbon emissions from the manufacturing sector by 86% (1098 MtCO_2_), i.e. from 1282 MtCO_2_ to 184 MtCO_2_ in 2050. Fig. 4 depicts the contribution of the associated component parts of each pillar to DDM, relative to potential emissions in 2050. The shares in the overall contributions from each pillar relative to potential emissions are:

- Pillar 1, *Energy Efficiency,* contributes 34%
- Pillar 2, *Material Efficiency,* contributes 22%
- Pillar 3, Industry-Specific Technologies, contributes 14%
- Pillar 4, *Power Grid Synergies,* contributes 30%

**Fig. 5.**
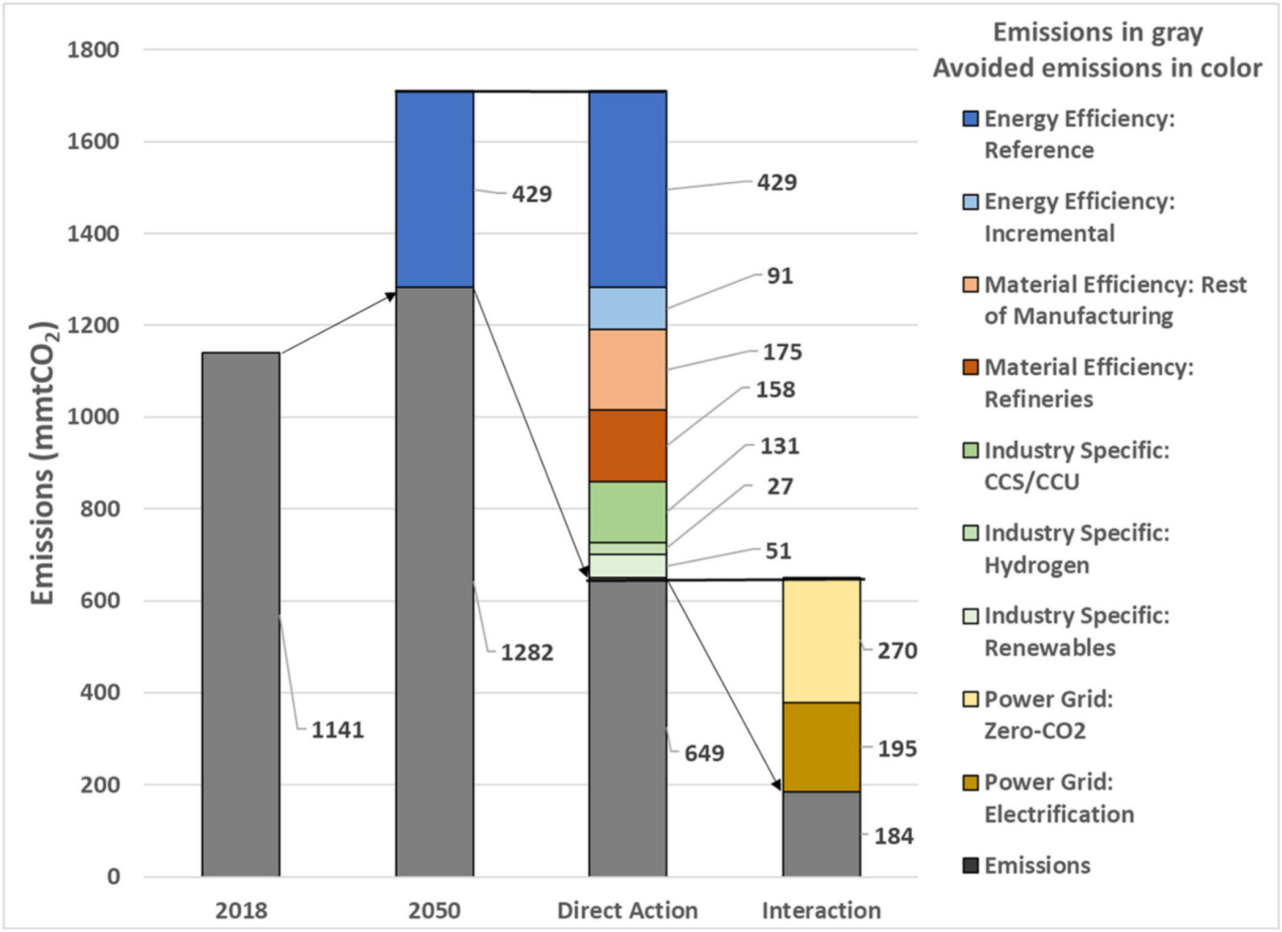
Summary of DDM Estimates by Pillar and Detailed Sub-Component (remaining emissions in grey, reductions in color).

Fig. 5 summarizes the estimates of DDM by each pillar relative to the base year of 2018 and potential emissions in 2050.^4^ Emissions are in grey and reductions, by pillar, are in color. The cumulative DDM estimates are *Direct action* (Pillars 1–3) and *Interaction* (Pillar 4).

**Fig. 6.**
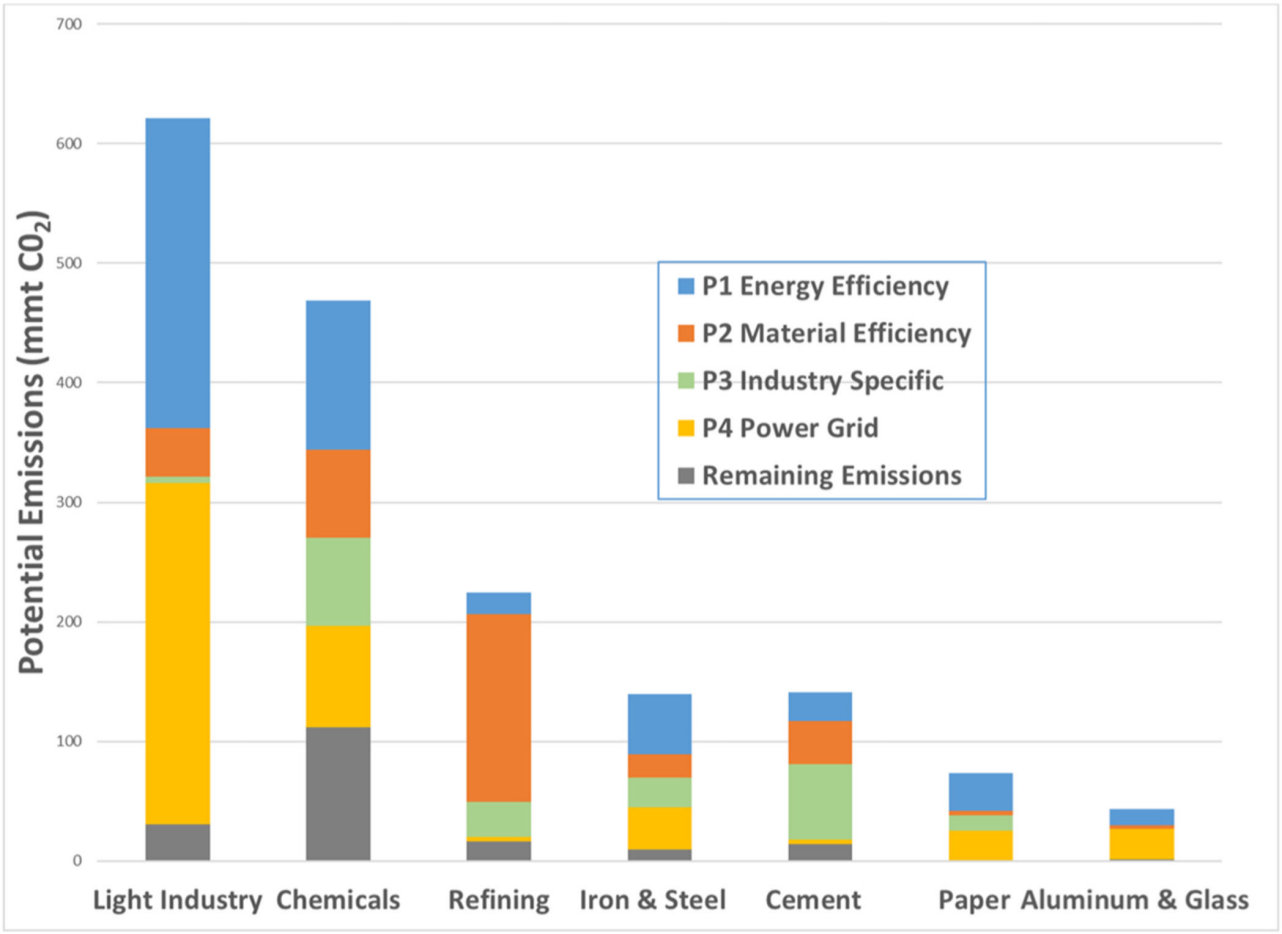
Industry-Specific Relative Contributions to DDM, by Type of Industry Action (remaining emissions in grey, reductions in color).

The study also estimates the industry-specific contributions to DDM. Fig. 6 shows the sector composition of the DDM reduction. Potential emissions in light industry are large and therefore comprise 39% of DDM emission reductions. This is followed by bulk chemicals (24%) and refining (14%). The other energy-intensive industries contribute the remaining 24% of DDM reduction. While individual sectors of light industry are small, relative to any single sector in heavy industry, collectively the higher economic growth rates of light industry (2.1% annual) vs heavy industry (1.1% annual) make light industry important to DDM by 2050 (see appendix for details). Within industries, we see that both light industry, aluminum and glass derive about one-half of their emission reductions from Pillar 4. In light industry, assembly and fabrication of good is generally more electricity intensive, but also the low-temperature heat demands on fossil fuels makes electrification attractive. Both of the underlying components of Pillar 4 are important to light industry. For glass and aluminum, the reasons are bi-partite; aluminum is very electricity intensive and benefits from grid decarbonization; glass has opportunities to convert furnaces from fuel to electricity. Cement and refining emission reductions come primarily from *industry direct action* for multiple reasons. The first is that material efficiency has a large impact in these industries. Reduction in gasoline demand will significantly lower refinery emissions. Reductions in clinker content of cement, combined with optimized construction practices will lower the overall production of this energy and carbon intensive process. While the optimization of material use is important for climate change mitigation (Haberl et al., 2020; Allwood et al., 2011), limiting growth is not sufficient to achieve (net) zero emissions by 2050; actual reduction of the demand may be necessary (Watari et al., 2020) and requiring rethinking growth-based strategies (see e.g. Hickel and Kallis, 2019). This study does not estimate zero emissions by 2050. This would require further reductions in material demand for cement or accelerated development of technologies that are currently not available. Grid purchased electricity is less important to the production process for both cement and refining. As about half of the emissions in cement come from process-related emissions, without reformulation of cement, or the reduction of cement demand, CCUS needs to play a dominant role as there are no other options we are aware of that can mitigate those process emissions.

Reductions in chemicals and iron & steel draw from a diverse set of actions in all four pillars, including both *Direct action* and *Interaction;* no single pillar dominates the emission reductions in either sector. Similar to light industry and paper, energy efficiency is a key enabler of reductions in both of these sectors. Material efficiency is also important, since the use of recycled feedstocks for plastics and steel are less carbon intensive. Improving the recovery and quality of those feedstocks will have its challenges, beyond the technology to process them. There are electrification options in these sectors as well. The EAF route for steel is well established for structural steel, but remains a challenge for sheet products. This is where hydrogen can play a role. In addition to recycled feedstocks, demand reduction as a decarbonization approach to material efficiency is also important in both sectors. Light weighting of product designs is important, as is consumer acceptance of elimination of single use products. It is noteworthy that Chemicals has the largest amount of remaining emissions in both absolute and percentage terms. The chemicals industry account for 64% of the remaining emissions. This suggests that additional focus on the chemicals sector will be needed for manufacturing to approach zero emissions without offsets, including the role of material efficiency, recycling, and the use of CCUS.

## 5. Conclusions

This paper presents an analysis of the opportunities for DDM in the United States. The estimates of CO_2_ emission reductions are relative to the well-established AEO 2019 Reference Case. The DDM estimates uses a detailed accounting exercise, using assumptions derived from other studies on a wide range of industrial sector-specific CO_2_ emission-reducing actions. The paper focuses in detail on eight industries: seven energy-intensive manufacturing industries, and one aggregate, non-carbon intensive “rest-of-manufacturing”. The paper identifies four pillars that support DDM: *Energy Efficiency*, *Material Efficiency*, *Industry-Specific*, and *Power Grid Synergies*. The first three pillars involve *direct industry action* and the last pillar involves *interaction* with the power grid to achieve emissions reduction. The DDM estimates presented reflect the direct or indirect nature of the actions.

The bottom-up analysis shows that an 86% reduction from the Reference Case in 2050 is feasible by implementing the low-carbon options comprised in the four pillars and that the reductions arise from a diverse suite of actions. Since industry is a heterogeneous sector it requires solutions tailored to specific processes and sub-sectors, which makes it hard to prioritize a single approach to achieve the goals of DDM. This paper illustrates that the 86% reduction is achievable by an “*all of the above”* suite of actions to reduce energy and selected process CO_2_ emissions from U.S. manufacturing, rather than focusing on a single or even small group of technologies. The heterogeneity of industry implies that, in aggregate, no single pillar dominates emission reduction; they all are needed to support DDM. Some individual sector reductions may rely more on one pillar, e.g. material efficiency in refining and cement and power grid synergies in aluminum, glass, and light industry. That is the nature of the industry-specific opportunities that are included in this study and the need for an industry-specific analysis. This paper does not identify a “silver bullet strategy” for DDM and illustrates what is achievable by the individual opportunities in the four pillars. The analysis highlights the diversity of measures to be included in a comprehensive strategy to mitigate the negative impacts of climate change. This is in contrast to other sectors, where a limited number of technologies may be sufficient to achieve deep decarbonization, e.g. electrification of light-duty vehicles for transport and decarbonization of the power grid using renewables, advanced nuclear, and/or CCUS of fossil fuels.

The analysis does show that energy efficiency of existing processes is a key enabler for deep decarbonization, providing various synergies, e.g. potential reductions in capital investments for energy conversion, grid development, and renewable power generation. Furthermore, material efficiency plays a key role in addressing the energy-intensive material producing industries. Unfortunately, this measure receives little attention in many energy and climate assessments (Worrell et al., 2016). Material demand in a climate-constrained world is key to develop sustainable climate policy.

In terms of electricity, onsite self-generation of renewable energy plays a key role for some industries, e.g. pulp & paper, food, but its role is limited for the total industrial sector. Electrification, in tandem with a rapidly decarbonizing and expanding power grid, shows important promise in a number of sectors. In the low-temperature energy services, electrification can lead to both energy and CO_2_ savings in a relatively short term. Selected high-temperature processes, e.g. electric furnaces in glass and metal industries, show promise for upscaling for the medium to long-term. This option does require large investments in new power generation and grid capacity, which would need immediate policy attention to support associated industrial decarbonization. However, industry could play a role in stabilizing the grid under a high penetration of intermittent renewables, by offering demand response and dispatchable power, e.g. food and forest industries.

Hydrogen and CCUS play a key role in selected industries, e.g. cement, steel, chemicals, where few alternatives are available. Both need large investments in infrastructure. Therefore, technology and infrastructure development should aim at these particular sectors.

This study does not project a pathway for industry to zero emissions. Despite the aggressive action needed to realize the potentials identified in this analysis, it shows that full decarbonization by 2050 may not be feasible using currently available technology. The feasible emissions reductions are large, yet 14% of emissions from manufacturing remain, with 2/3rds of these emissions in the chemicals sector. This implies that additional focus is necessary on the chemicals sector, including increased attention for material efficiency, recycling, bio-based feedstocks and the use of CCUS. Radical new technologies, e.g. electrochemistry, that acknowledge a future not built on fossil feedstocks and thermal energy will be important on the long-term in this industry. The remaining emission reductions to come to net zero CO_2_ emissions would need to come either from offsets, additional future reduction measures beyond those included in the analysis, or a combination of both. Full decarbonization of industry is only feasible if the need for growth in the production of energy-intensive materials results in a reduction of total material demand and production. This requires not only policies to limit material use, including the potential of rebounds, but also a more fundamental re-evaluation of economic growth as a driver for human welfare.

The paper’s estimates makes simplifying assumptions that the 30-year time horizon which would substantially, or completely, result in the turnover of the energy-using capital stock in manufacturing. This complete replacement assumption implies, ceteris paribus, that any *delay in implementation will result in higher emissions or higher cost* due to early retirement of capital later in the next three decades. Companies that are examining how they can contribute to decarbonization can use these industry-specific results as a roadmap to begin evaluating future strategies. A strategy building on diversity of actions has the advantage that some measures can be applied immediately, e.g. efficiency, electrification and decarbonization of power supplies. Other actions may require broader societal adaptions, like increased recycling and emerging technology, such as CCUS.

This study does not consider the possible impact of rebound on emission reductions from Pillar 1, Energy Efficiency, which is over 1/3rd of reductions. Rebound can occur when an energy service becomes cheaper due to efficiency and there is a positive elasticity of demand for that service. Other mechanisms for rebound include economy wide impact on the demand for goods and services. In their review of the literature, Sorrell and Dimitropoulos (2008) have found a lack of consensus with regard to a consistent method to measure the rebound effect and that many studies overestimate their impact. However, energy efficiency for DDM can reduce the cost of goods and services produced by manufacturing and become a source of economy wide rebound. If a carbon price is a policy option to motivate DDM, then those costs may, or may not, experience a net decline under both a carbon price and energy efficiency. Whether a carbon price might mitigate concerns over rebound or not, it should be made clear this study does not incorporate rebound on our estimates.

DDM will only occur if reducing CO_2_ emissions to avoid climate change is a priority. Given the *all of the above* nature of the DDM estimates presented in this paper, we expect that an *all of the above* policy environment will also be required to motivate change and lower barriers to implementation. Support for technical and managerial challenges, complementary federal/state/local voluntary programs, targeted investment credits/rebates, and carbon pricing will all likely be needed to manage the systemic transition of the magnitude the paper envisions.

The cost effectiveness of some of these actions in light of future economic conditions requires study. The opportunities to reduce primary material production needs attention within a comprehensive climate policy agenda that has often focused on energy supply. The role that industry might play in grid decarbonization deserves more consideration, e.g. to provide demand response for intermittent renewables or with purchase power agreements. Additional study will be needed on the relative merits of different specific polices, including the various forms of carbon pricing, on the manufacturing sector.

Finally, the study serves as an example for the analysis of deep reductions of industrial GHG emissions for other countries. It helps to understand the diversity in technological opportunities and how they can be combined to build a menu for deep reductions.

## Supplementary Material

Appendix A

## Figures and Tables

**Table 1:** Summary of technical opportunities for emission reduction in selected industry sectors or subsectors.

Pillar		Pulp & Paper	Iron & Steel	Chemicals	Cement & Lime	Petroleum Refining	Aluminum	Glass	Light Industries

P1	Energy efficiency	40% efficiency improvement in fuel demand (of which 16% included in Reference case) 30% savings on electricity (of which 23% is included in Reference case).	Energy efficiency improvement by switching to Best Available Technology (BAT) of 39%	Potential energy savings of 19% with currently available technology Potential savings of 31% with advanced technology	Potential for energy efficiency improvement with current technology at 34%, A further 4% potential savings with technology currently under development	Potential of 14% efficiency improvement with current technology, 26% additional energy savings with technologies that are in various stages of R&D	Limited potential of about 10% beyond the energy savings in the reference case.	Additional potential is estimated at 33% Technologies under development could add another 9% savings	Potential savings of 25% for fuel end uses, and 30% for electric end uses
P2	Material Efficiency	No demand reduction, as material efficiency is offset by a move away from plastics Increased use of *recycled fiber* from 37% to 56% by 2050 will reduce fuel demand by 15%	Share of recycled steel increases to 80% by 2050 Iron production declines to 16 Million mt/year	Increased material efficiency in product design and recycling varies from 7% up to even 55% Plastic recycling decreases the energy used to make plastic by 25%–55%	In line with global IEA scenarios, we assume that the clinker-to-cement ratio can be reduced to 70%, from 92% now	In line with the IEA gasoline/diesel demand will decrease by 70% by 2050.	Using part of the exported scrap domestically allows primary smelter production to decrease by about 25%	About 11 mt of glass waste is produced in the United States. Increasing the recycling rate would reduce emissions by 2 mmtCO_2_	Demand will be reduced by 10% (on average) across all other industries
P3	Renewables	Biomass-based CHP units increase efficiency allowing all integrated mills to operate fully on renewables 15% use of renewables in stand-alone paper mills	The share of renewables in the steel industry will be very limited due to process requirements.	Up to 15% savings due to shifting to biomass-based feedstocks Other forms of direct use of renewable energy provide up to 5% of energy	Up to 30% use of biomass fuels	Biofuels offset up to 15% of refinery production. We do not assume further internal use of renewable energy	No specific opportunities identified	No specific opportunities identified	25% of heat demand can be met by renewables (e.g. in food industries)
	Hydrogen	No value of Hydrogen use	Half of remaining iron production is replaced by hydrogen-based DRI-production.	In line with The IEA estimates hydrogen could reduce CO_2_ emissions by 10% in 2050	No application of hydrogen assumed in lime and cement kilns	Furnaces in a refinery can be fired with (self-generated) hydrogen	Limited potential for furnaces to be converted to use hydrogen. Electrification may be more attractive.	Hydrogen could be used as fuel. Electrification may be more attractive.	Hydrogen would be a less attractive compared to electrification and renewables
	CCUS	No use of CCUS due to location mills.	Half of remaining iron production is produced in smelt reduction plants with CCUS (resulting in emission reductions of 80–90% compared to current primary production process)	CCUS to contribute to an emission reduction of 20% of emissions	CCUS using calcium- looping would reduce emissions by 90% CO_2_ curing of concrete may reduce emissions by 300 kg/mt cement	Centralized CCUS from hydrogen plant used as internal fuel	No role	No role	No role
P4	Electrification	Electrification leads to a 22% emission reduction (half from electric boilers, and the other half shared between heat pumps and direct electric drying).	Electrification due to increased use of electric arc furnaces (EAFs) and electric furnaces (induction or plasma)	Up to 20%–25% of current fuel use could be replaced by electric heating	Too early to evaluate the feasibility of this application for the US	Electrification is not attractive	For primary smelters, electricity is already the key energy source. Heating furnaces can be electrified (as some are already).	Full electrification of large furnaces possible by 2050	50% reduction of fuel use in heat (30% by electric boilers and 20% from heat pumps or Mechanical Vapor Recompression).

## Footnotes

1 In a global context, “net zero” refers to balancing any amount of emitted greenhouse gases with equivalent emissions that are sequestered. This differs from a building/facility-level notion of “net zero” where offsets of emission reductions are allowed elsewhere.

2 On-site renewable power generation may occur in manufacturing, with some additional benefits of lower transmission and distribution losses, but those are not included in this pillar.

3 https://www.energystar.gov/buildings/tools-and-resources.

4 Recall that potential emissions are defined as Reference Case emissions plus the avoided emissions from energy efficiency included in the Reference Case.

## Table of abbreviations/nomenclatures

AEO: Annual Energy Outlook
BAT: Best Available Technology
CCU: Carbon Capture And Utilization
CCUS: Carbon Capture, Utilization & Storage and Utilization
CHP: Combined Heat and Power
CO_2_: Carbon Dioxide
DDM: Deep Decarbonization of Manufacturing
DRI: Direct Reduced Iron
EAFS: Electric Arc Furnaces
EIA: Energy Information Administration
GHG: Greenhouse Gas
HBI: Hot Briquetted Iron
HVAC: Heating Ventilation and Cooling
IR: Infrared
NZA: Net Zero America
U.S.: United States
ZCAP: Zero Carbon Action Plan
