# Supplementary material for: Bottom-up estimates of deep decarbonization of U.S. manufacturing in 2050
Source: J Clean Prod. Author manuscript; Available in PMC 2022 Sep 6. (PMC9446384; doi:10.1016/j.jclepro.2021.129758)
Supplement: Appendix A [file NIHMS1767417-supplement-Appendix_A.docx]

# Appendix: Supporting information

Summary of AEO2019 Industrial Forecast Used in the Reference Case

In aggregate, total source energy use in industry grows at 0.8% per year over the study period. Fuel use grows at 1.0% per year and purchased (site) electricity grows at a similar rate (0.9% per year), but improvements in the power generation sector include lower losses due to improved power plant efficiency and penetration of PV. Source electricity, including losses, grows at only 0.4% per year. However, total industry production (value of shipments) grows at 1.8% per year, with manufacturing growing at 1.9% annually. This implies that total energy intensity is improving at about -1.0% per year. Fuel intensity and purchased (site) electricity intensity both improve at similar rates. This implies that in the Reference Case, at least in aggregate, there is no trend toward electrification in industry. However, the lower growth rate in total source energy intensity (0.8% vs. 0.9–1.0% per year) arises from changes in the power sector, not the industrial sector. Natural gas remains the single largest type of energy consumed for heat and power, growing at approximately the same rate as total source energy consumption, implying no shift in total manufacturing fuel mix.

The AEO Reference Case projects direct energy related manufacturing plant emissions (Scope 1) for each industry.^[[1]](#footnote-2)^ Total emissions from power generation are projected separately. To get indirect emissions from manufacturing plant electricity use (Scope 2), the study allocates the Reference Case power sector emissions based on the share of electricity use in each industry.^[[2]](#footnote-3)^ Industry-wide total energy-related CO_2_ emissions (direct plus indirect) grow at 0.5% per year. This is composed of a 0.7% increase in direct fossil fuel emissions and a decline in indirect emissions attributable to electricity use, since the aggregate power sector CO_2_ emission rate is improving at a rate of -1.0%. This is without any additional climate policy changes and assumes existing laws will remain in place or expire as currently written. It should be noted that, in the Reference Case, economy-wide energy-related CO_2_ emissions are basically flat. This means that declines in emissions from transportation and residential and commercial buildings offset the 0.5% annual growth from industry.

Looking at the underlying economic activity, the manufacturing portion of industry is a fundamental driver for energy use; energy-intensive (“heavy”) industries are forecast to grow more slowly (1.1% per year) than non-energy-intensive (“light”) industries (2.1% per year). There is substantial variation in growth, with chemicals and cement growing the most and metals least. Refinery output declines on a dollar basis but remains flat in terms of physical barrels because prices for refinery products are projected to decline slightly.

Fossil fuel use also grows more slowly in energy-intensive industry, at 0.3% vs. 0.8% annually in non-energy-intensive industry. Fossil energy intensity declines more quickly for light industry (-1.3% per year) vs. heavy (-0.5% per year). The share of total fossil fuel use in heavy industry only shifts 3% relative to light industry. Electricity use in heavy industry grows at a similar rate to fossil fuel use, but electricity use in light industry grows faster (1.3% per year) than fuel use (0.8% per year). This should not be taken as evidence of electrification *per se* in light industry. It is likely a combination of the relative growth of industries that use more electricity, rather than fuel switching. CO_2_ emissions also grow slightly more quickly in light industry than heavy industry (0.8% vs. 0.5% annually), with the share of CO_2_ emissions from heavy industry remaining fairly stable.

Since the Reference Case includes the response to energy prices, ongoing policies, trends, and new technology adoption, this results in improved energy efficiency. This study computes the level of energy efficiency that is accounted for in the Reference Case as the different between energy use in 2050 based on a “*frozen efficiency*” assumption where energy grows proportionally to industry shipments and the Reference Case level that has lower energy growth due to efficiency effects. This can be viewed as a counterfactual, “no-action scenario,” representing an upper bound estimate of ***potential emissions*** attributable to industry growth. We are explicit about whether the reported emission reductions are relative to the Reference Case or the upper bound of ***potential emissions***, with the difference being energy efficiency that is embodied in the Reference Case.

The amount of efficiency that is included in the Reference Case varies by industry, from a low of 4% to a high of 34%; ~19% for heavy industry and ranging from 22-34% for light industry. When developing our estimates, we consider the potential for energy efficiency based on other sources (see below) and compare them to the energy efficiency that is implicit in the Reference Case. If the industry-level studies suggest higher potential savings, then the difference from what is included in the Reference Case and the study estimates is included as ***incremental energy efficiency***. In other words, there is a substantial amount of energy efficiency (Pillar 1) included in the Reference Case, so the energy efficiency estimates include the incremental efficiency, in addition to the energy efficiency already accounted for in the Reference Case.

Table A: Emission reductions by industry and pillar, relative to potential emissions in 2050 (mmt CO_2_)

|  | Light Industry | Bulk Chemical | Refining | Cement | Iron & Steel | Paper | Aluminum & Glass |
| --- | --- | --- | --- | --- | --- | --- | --- |
| Potential Emissions | 621 | 469 | 224 | 141 | 140 | 73 | 43 |
| P1 Energy Efficiency | 259 | 125 | 17 | 24 | 51 | 31 | 13 |
| Reference | 221 | 103 | -1 | 23 | 45 | 24 | 13 |
| Incremental | 38 | 22 | 18 | 1 | 6 | 7 | 0 |
| P2 Material Efficiency | 40 | 73 | 158 | 37 | 19 | 4 | 3 |
| P3 Sector Specific | 5 | 74 | 29 | 63 | 25 | 12 | 0 |
| Renewables | 5 | 28 | 0 | 6 | 0 | 12 | 0 |
| Hydrogen | 0 | 19 | 0 | 0 | 8 | 0 | 0 |
| CCUS | 0 | 28 | 29 | 57 | 17 | 0 | 0 |
| P4 Power Grid Synergy | 286 | 85 | 4 | 4 | 35 | 26 | 26 |
| Zero Carbon grid | 164 | 38 | 4 | 4 | 28 | 19 | 13 |
| Electrification | 122 | 46 | 0 | 0 | 7 | 7 | 13 |
| Remaining Emissions: | 31 | 112 | 16 | 14 | 10 | 0 | 1 |

1. For detailed definitions of Scope 1, 2, and 3 emissions, see https://ghgprotocol.org/sites/default/files/standards_supporting/FAQ.pdf [↑](#footnote-ref-2)
2. Scope 3 emissions are only relevant from a carbon footprinting exercise. This study captures all direct and indirect (scope 1 and 2) emissions, but no emissions from product use or material content. The later component is already accounted for in the emissions of upstream, primary materials industries. [↑](#footnote-ref-3)
